# Quality of Life in the First Year of Follow-Up in a Randomized Multicenter Trial Assessing the Role of Imaging after Radical Surgery of Stage IIB-C and III Cutaneous Melanoma (TRIM Study)

**DOI:** 10.3390/cancers14041040

**Published:** 2022-02-18

**Authors:** Ylva Naeser, Hildur Helgadottir, Johan Hansson, Christian Ingvar, Nils O. Elander, Petra Flygare, Cecilia Nilsson, Frida Jakobsson, Antonios Valachis, Dimitrios Papantoniou, Agneta Nordin Danfors, Hemming Johansson, Anders Sundin, Yvonne Brandberg, Gustav J. Ullenhag

**Affiliations:** 1 Rudbeck Laboratory, Department of Immunology, Genetics and Pathology, Uppsala University, 751 85 Uppsala, Sweden; ylva.naeser@igp.uu.se; 2 Department of Oncology, Uppsala University Hospital, Entrance 101, First Level, 751 85 Uppsala, Sweden; 3 Department of Oncology-Pathology, Karolinska Institutet, 171 77 Stockholm, Sweden; hildur.helgadottir@regionstockholm.se (H.H.); hemming.johansson@ki.se (H.J.); yvonne.brandberg@ki.se (Y.B.); 4 Theme Cancer, Karolinska University Hospital, 171 76 Stockholm, Sweden; johan.hansson@ki.se; 5 Department of Surgery, Clinical Sciences, Lund University, BMC F12, 221 84 Lund, Sweden; christian.ingvar@med.lu.se; 6 Department of Biomedical and Clinical Sciences, Linköping University, 581 85 Linköping, Sweden; nils.elander@liu.se; 7 Department of Oncology, Sundsvall County Hospital, Lasarettsgatan 21, 856 43 Sundsvall, Sweden; petra.flygare@rvn.se; 8 Department of Oncology, Hospital of Västmanland Västerås, 721 89 Västerås, Sweden; cecilia.nilsson@regionvastmanland.se; 9 Department of Oncology, Örebro University Hospital, 701 85 Örebro, Sweden; frida.jakobsson@regionorebrolan.se; 10 Department of Oncology, Faculty of Medicine and Health, Örebro University, 701 82 Örebro, Sweden; antonios.valachis@oru.se; 11 Department of Oncology, Ryhov County Hospital, 551 85 Jönköping, Sweden; dimitrios.papantoniou@rjl.se; 12 Department of Oncology, Visby County Hospital, St. Göransgatan 5, 621 84 Visby, Sweden; agneta.nordin-danfors@gotland.se; 13 Department of Surgical Sciences Radiology & Molecular Imaging, Uppsala University, 751 85 Uppsala, Sweden; anders.sundin@radiol.uu.se

**Keywords:** melanoma, follow-up studies, positron emission tomography computed tomography, tomography, X-ray computed, quality of life, randomized controlled trial, prospective studies

## Abstract

**Simple Summary:**

After surgery of high-risk melanoma, patients are usually followed up by physical examinations. Recommendations regarding imaging vary due to insufficient evidence of the benefit of regular scans. It might also be stressful for patients to undergo imaging. In an ongoing Swedish study, half of the patients are randomized to whole-body imaging in addition to physical examinations. Three imaging procedures are performed during the first year. The main aim of our study was to investigate if imaging during the first year of follow-up affected the patients’ well-being. Validated self-reporting questionnaires regarding symptoms of anxiety and depression and quality of life were answered at study start and at 1 year. Questionnaires from 204 recurrence-free patients were analyzed. No differences in either level of anxiety/depression or quality of life were found at 1 year follow-up between the imaging and non-imaging group. These findings can be considered in the formulation of future follow-up programs.

**Abstract:**

The benefit of imaging in the follow-up setting for high-risk melanoma patients is uncertain, and even less is known about the impact of intensive follow-up on the patient´s quality of life. In 2017, a Swedish prospective randomized multicenter study started, in which high-risk melanoma patients are randomly assigned 1:1 to follow-up by physical examinations +/− whole-body imaging. The first-year examinations are scheduled at 0, 6 and 12 months. The aim of this study was to investigate whether the patients´ health-related quality of life (HRQoL) and levels of anxiety and depression were affected at 1 year by imaging. Anxiety/depression and HRQoL were assessed at 0 and 12 months by the questionnaires Hospital Anxiety and Depression (HAD) scale and EORTC QLQ-C30 version 3. Expected baseline QLQ-C30 values for the patients were calculated using data from the general population. In total, 204 patients were analyzed. Mean differences in subscale scores at 1 year were not statistically significant either for HRQoL or for anxiety/depression. Baseline HRQoL did not differ from expected values in the general Swedish population. In conclusion, the patients in general coped well with the situation, and adding whole-body imaging to physical examinations did not affect the melanoma patients’ HRQoL or levels of anxiety or depression.

## 1. Introduction

The incidence of cutaneous melanoma is rapidly increasing in many countries including Sweden. Consequently, a growing number of patients are enrolled in follow-up programs following primary treatment. Primary treatment consists of surgery with a wide excision of the primary tumor with a margin of 1–2 cm, depending on the depth of growth and location of the melanoma. A sentinel node biopsy is usually recommended if the primary melanoma is thicker than 1 mm [1]. Due to lack of evidence on how a follow-up program should be optimally composed, national guidelines for follow-up after radical surgery for melanoma vary substantially. Although no randomized study has so far been conducted, some studies indicate earlier detection of distant and/or loco-regional recurrent disease for high-risk melanoma when imaging is added to follow-up by physical examinations [2,3,4]. High-risk melanoma, i.e., melanoma with high-risk of recurrent disease after primary surgery, is often defined as post-operative stage IIB, stage IIC and all stage III. Stage IIB-C corresponds to a thickness of 2–4 mm with ulceration and all melanomas thicker than 4 mm. Stage III includes loco-regional lymph-node spread and in-transit and satellite metastasis [5].

Early detection of recurrent disease might be beneficial because the treatment landscape has changed in the past decade and newer treatments such as immunotherapy and targeted therapies seem to have a better outcome if tumor burden is low [6,7]. Hence, the role of imaging as part of routine follow-up is unclear. Nonetheless, there is an international tendency towards more intense follow-up programs including imaging. According to UK and Australian guidelines, regular imaging with FDG-PET/CT, CT and/or MRI during follow-up may be considered [8,9]. Another example is Denmark, where PET/CT is recommended during follow-up for high-risk patients [10]. In a recently published cohort study in patients with stage IIB–III, routine PET/CT led to the detection of recurrent disease in 18% of the patients [11].

Swedish national guidelines do not recommend whole-body imaging in the follow-up setting, except for one examination before and at 6 months after starting adjuvant systemic therapy [12].

Drawbacks with imaging in the follow-up setting include costs and other resources required, radiation exposure and risk for decreased renal function. Other potential side-effects are mental symptoms and a negative impact on patients´ health-related quality of life (HRQoL), even when no recurrent disease is diagnosed. The waiting periods before and shortly after imaging, i.e., before results have been presented to the patient, and false-positive results are possible reasons for these latter potential drawbacks. For example, in an Australian prospective cohort study, 154 patients with resected stage III melanoma underwent regular CT or FDG-PET/CT at a minimum of every 6 months; false-positive results and incidental findings were identified in 81 patients (53%), leading to additional tests, referrals and procedures. Of note, HRQoL was not assessed in this study [13]. On the other hand, patients may feel more reassured with a more extensive follow-up, especially when imaging findings are normal. Studies of the impact of follow-up programs on patients´ HRQoL, are rare, and especially considering routine imaging. To our knowledge, no prospective studies have previously been conducted to address this question in melanoma patients.

In 2017 a prospective randomized multicenter study started in Sweden, the Trial to assess the Role of Imaging during follow-up after radical surgery of stage IIB-C and III cutaneous malignant Melanoma (TRIM study), with overall survival at 5 years as the primary endpoint. One of the secondary endpoints is HRQoL.

The aim of this study was to investigate whether there is a difference in HRQoL at 1 year between patients undergoing high-intensity scheduled follow-up applied in the TRIM study protocol, and those followed according to the Swedish national guidelines.

## 2. Patients and Methods

### 2.1. Patients

The study protocol for the TRIM study has previously been described in detail [14]. The main inclusion criterion is radical surgery for stage IIB-C or stage III cutaneous melanoma. Key exclusion criteria are: comorbidity or general condition prohibiting treatment in case of recurrent disease, current or previous malignancy within 5 years, and participation in other clinical studies interfering with the follow-up program. Written informed consent was obtained from all patients before inclusion in the study. The study was approved by the regional Ethics Board of Uppsala, number 2017/028. Patients were included at 10 oncology sites between June 2017 and January 2020. Randomization was carried out directly at site in the electronic data capture system (Viedoc) after receipt of written informed consent. Patients were stratified according to tumor stage and radiological assessment method. Included patients were randomly assigned 1:1 to 3 years of follow-up according to current Swedish national guidelines (arm A) or according to guidelines plus whole-body imaging and blood test including tumor marker S-100B (arm B) at baseline and at 6, 12, 24 and 36 months.

### 2.2. Quality of Life Related Assessments and Instruments

Methods for assessing the effects on HRQoL by symptoms of disease, adverse effects of treatments and procedures in cancer patients usually include patient-reported outcomes (PRO). A well-established instrument is the cancer-specific questionnaire QLQ-C30, version 3, provided by the European Organisation for Research and Treatment of Cancer [15]. For assessment of levels of anxiety and depression in somatically ill patients, the Hospital Anxiety and Depression (HAD) scale is widely used [16,17].

HRQoL and anxiety/depression were assessed using the aforementioned validated questionnaires at baseline and at 12, 24 and 36 months for patients included at oncology sites. HRQoL- and HAD questionnaires were obtained at baseline, after informed consent but before randomization, and at the 12-month follow-up visit. Questionnaires at 12 months were completed according to protocol, either with a web-based device or paper format at site before doctor´s appointment. In this study, data from baseline visit and 1-year follow-up are reported.

EORTC QLQ-C30 Version 3 is a cancer-specific questionnaire consisting of 30 items including five functional scales (physical function (PF), role function (RF), emotional function (EF), cognitive function (CF) and social function (SF)), three multi-item symptom scales (fatigue, pain, nausea and vomiting); and six single items (dyspnea (DY), loss of appetite (AP), constipation (CO), insomnia (SL), diarrhea (DI), and financial difficulties related to disease (FI)); and one scale on global health and overall quality of life (QL). Most items are responded to on a 4-point scale ranging from 1 “Not at all” to 4 “Very much”. The two items assessing global health and QL are responded to in seven categories ranging from 1 “Very poor” to 7 “Excellent”. Higher scores represent better functioning on the functioning subscales and overall health and quality of life, but more symptoms or problems on the symptom scale items. Reference values from the Swedish population are available [18].

The Hospital Anxiety and Depression (HAD) scale consists of 14 items: 7 assessing anxiety and 7 assessing depressive symptoms. Every item is graded 0–3, where 0 represents “no problem”. Each subscale is summed, giving a maximum of 21. Two cut-off points have been suggested: 0–7, representing no problems of clinical relevance; 8–10, for cases that warrant further psychiatric investigations (possible cases); and 11–21, indicating clinical levels of anxiety/depression (probable cases). The Swedish version has been validated in patients with melanoma [19].

### 2.3. Statistics

The values on the EORTC QLQ-C30 subscales were calculated and transformed to a 100-graded scale according to guidelines [20]. Clinically meaningful changes in EORTC QLQ-C30 have previously been established [21]. A difference of 5–9 points is considered as small, 10–19 as moderate, and more than 20 as large. Analyses of the EORTC QLQ-C30 and HAD subscales were performed by using linear mixed models with center as a random effect and including baseline scale scores in the models. Results from these models are presented as mean differences together with 99% confidence intervals (CI) between the study arms at 1 year. The expected mean scale scores at baseline, for both arms combined, were calculated using indirect standardization with age- and sex-adjusted normative scores from the general Swedish population.

Fisher´s exact test was used to compare the study arms regarding number of patients at normal levels and possible and probable cases for anxiety and depression, respectively. The level of significance was set to 1% to take multiple testing into account. For all statistical analyses, StataCorp. 2021 Stata Statistical Software (Release 17, StataCorp LLC, College Station, TX, USA) was used.

## 3. Results

### 3.1. Characteristics of Included Patients

**Figure 1:**
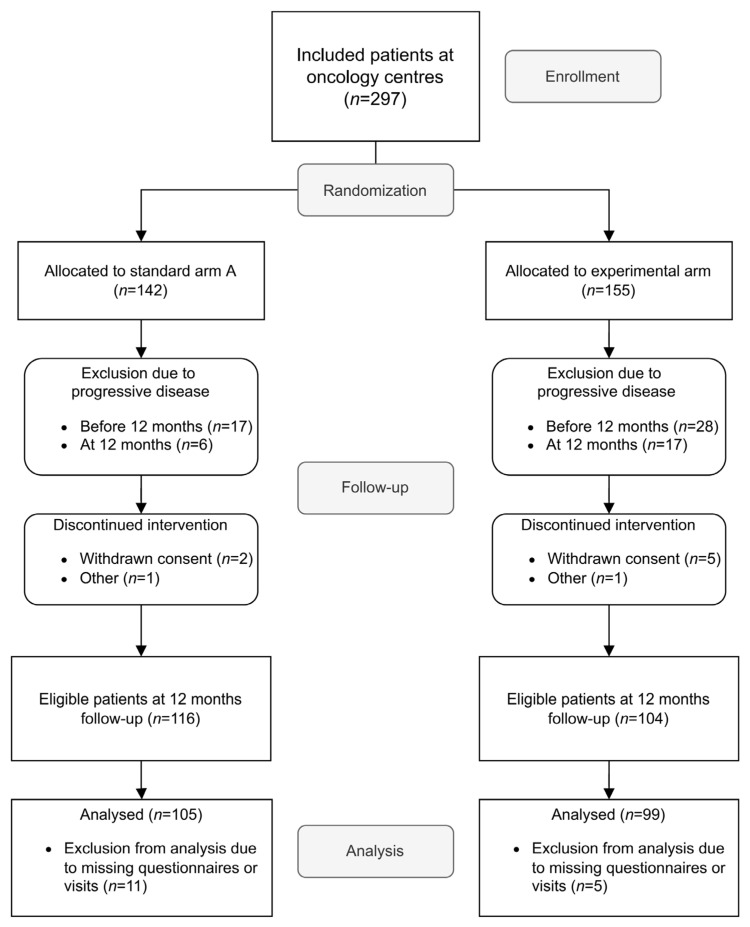
CONSORT diagram. Patients included at all TRIM-study oncology sites between June 2017 and 31 January 2020 were considered eligible for inclusion. Sites: Four university hospitals (Uppsala, Karolinska, Linköping and Örebro) and six county hospitals (Västerås, Jönköping, Eskilstuna, Gävle, Sundsvall and Visby).

A total of 297 patients were included at the 10 oncology sites in the TRIM study during the study period. Patients were excluded due to the following reasons: relapse of melanoma before (*n* = 45) or at 1-year follow-up (*n* = 23), withdrawn consent (*n* = 7), or other reason (*n* = 2). Details on the reasons for exclusion per study arm are shown in Figure 1. Thus, 220 patients (arm A = 116, arm B = 104) were considered eligible for analysis. Sixteen patients could not be analyzed due to missing questionnaires at baseline or 1-year follow-up, or because the follow-up visit at 1 year was not performed according to protocol. A total of 204 patients were included in the final analysis (Figure 1).

Arm A (standard arm) included 105 patients and arm B (experimental arm) included 99 patients. Patients in arm B had a median age of 61 years, and in arm A the median age was 67 years. In both arms, about 60% were men and the majority were diagnosed with Stage III melanoma (A = 53 %, B = 59%). Approximately 15% of patients had undergone a complete lymph node dissection, and about 20% received adjuvant systemic therapy after surgery. Only three patients in arm B and no patients in arm A received adjuvant radiotherapy. Overall, the clinical features in the two study arms were similar. Patient characteristics are listed in detail in Table 1.

### 3.2. Health Related Quality of Life (HRQoL) and Anxiety/Depressive Symptoms

**Figure 2:**
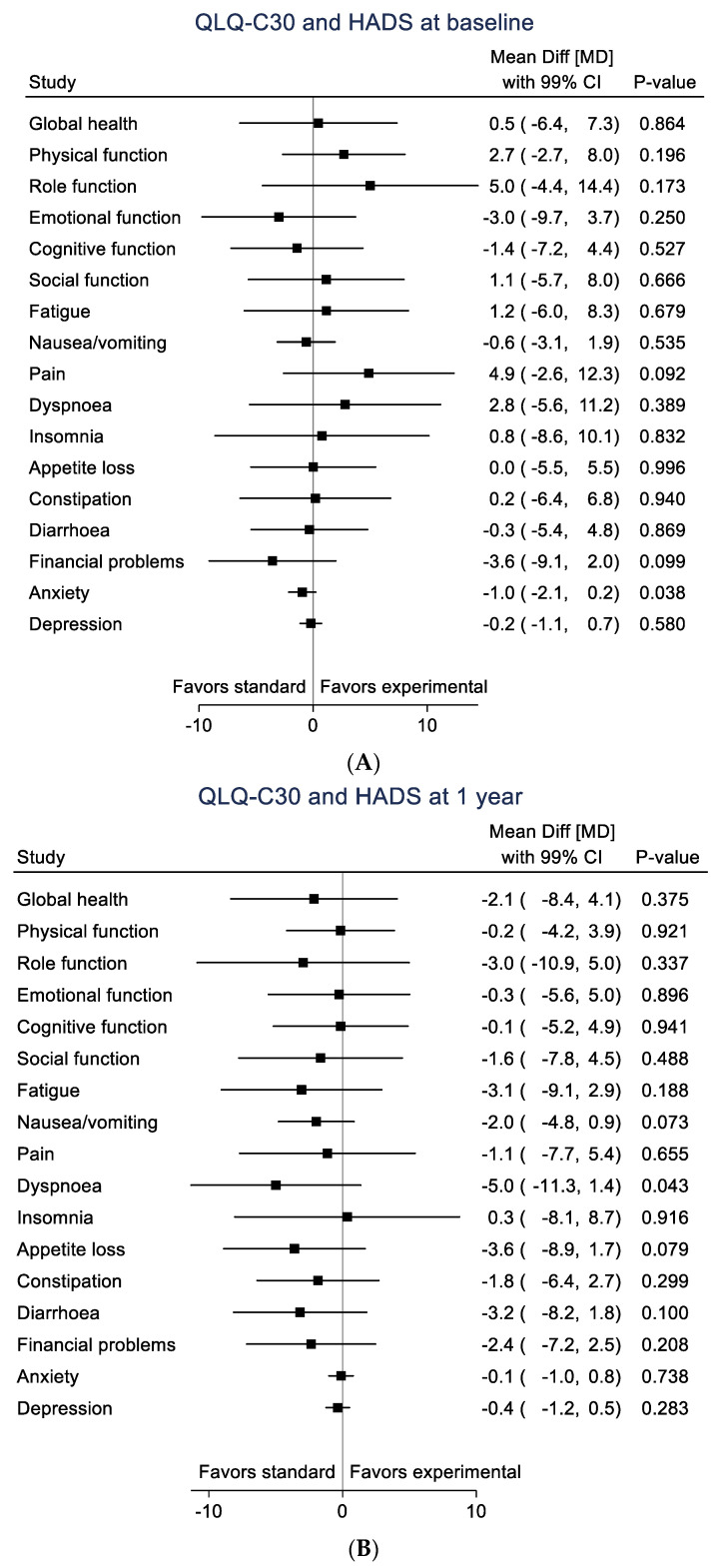
Black squares represent mean differences for each subscale with 99% CI. (**A**). QLQ-C30, HAD-A and HAD-D at baseline. (**B**). QLQ-C30, HAD-A and HAD-D at 1 year.

No statistically significant differences were found in either HRQoL subscales or level of anxiety/depressive symptoms (HAD scale) between the two study arms either at baseline or at 1-year follow-up (Table 2 and Figure 2A,B).

**Figure 3:**
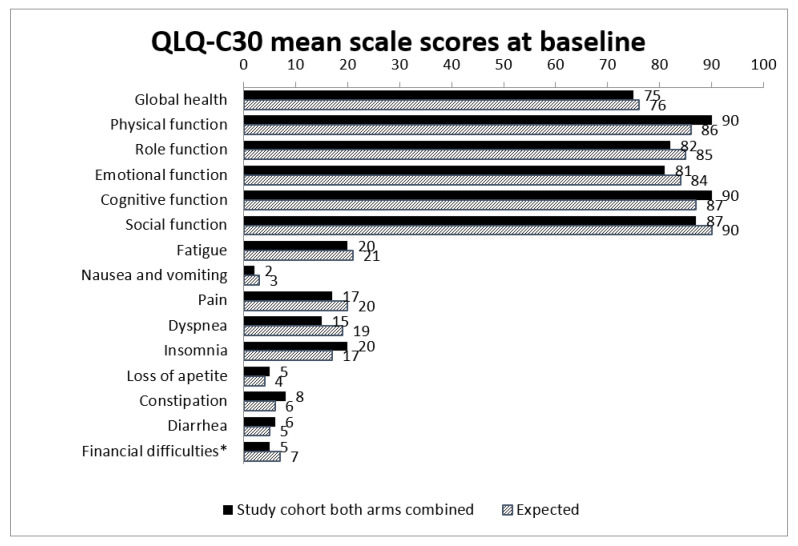
Observed and expected mean scale scores for both arms combined at baseline compared to general population (expected). Expected mean scale scores are calculated using indirect standardization with normative scores from the general Swedish population. * Financial difficulties related to disease.

Moreover, neither at baseline nor at the 1-year follow-up were there any statistically significant differences in HRQoL subscales between the total study group and expected values in the general population when comparing baseline data to age- and sex-adjusted normative data for the Swedish population (Figure 3).

**Figure 4:**
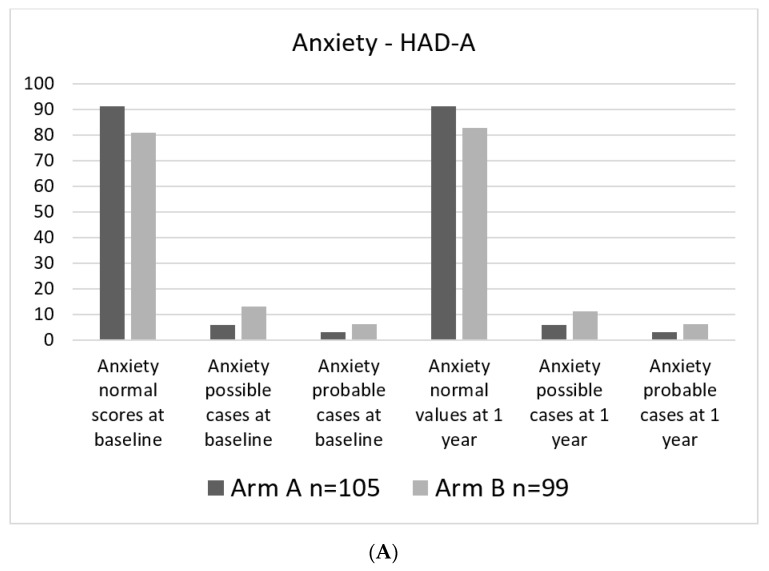

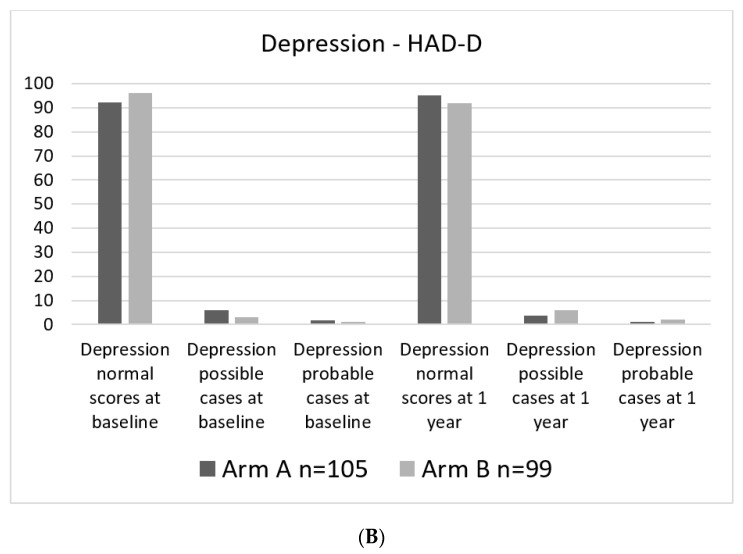
Percent of patients in each category. (**A**). HAD-A categorical at baseline and 1 year. (**B**). HAD-D categorical at baseline and at 1 year.

Levels of anxiety and especially depressive symptoms were generally low in the study group. At baseline assessments, 1.9 and 1.0% of study participants, respectively, were considered probable cases regarding depressive symptoms. Levels of anxiety at baseline were higher, with 6.1% probable cases in the experimental arm and 2.9% probable cases in the standard arm. (Figure 4A,B).

## 4. Discussion

The TRIM study, as previously described, aims to investigate whether high-risk melanoma patients will benefit from the addition of whole-body imaging and regular blood tests as compared to the Swedish routine follow-up schedule. One potential effect of an intense follow-up program is a negative impact on patients’ well-being because the procedures and the waiting period before receiving results of investigations might be stressful, but could also be comforting.

In our study we did not find any statistically significant differences, in HRQoL or regarding depressive symptoms or anxiety levels between the two study arms at 1-year follow-up. As expected, there was a higher rate of recurrence in the experimental arm during the first year, and thus, numerically more patients not eligible for analysis. This difference is unlikely to have had an impact on the final analysis.

The reasons why the intensity of the follow-up schedule did not affect the HRQoL or levels of depressive and anxiety symptoms are unclear. It is plausible that the patients felt equally reassured, or equally worried, merely from being part of a follow-up program and with imaging procedures constituting only one part of the whole follow-up management. One might also speculate that increased care by regular imaging may on one hand entail a positive effect on the patients´ HRQoL and decrease depressive and anxiety symptoms, but on the other hand, may impose discomfort during the waiting period for the scanning results, which to a similar degree may increase symptoms of depression and anxiety. Fast reading and reporting routines for the imaging procedures are, therefore, likely to shift this balance towards the positive side. The most important factor influencing HRQoL, levels of depressive symptoms, and anxiety could have been the doctors´ appointments, which were carried out in the same way and at equal intervals in both study groups.

One interesting finding was that HRQoL of the study participants at baseline was similar to that in the Swedish normal population, adjusted for age and sex. These results indicate that patients manage to cope well and were able to fully and rapidly adjust to the new situation. This finding is consistent with the results from the Nordic adjuvant interferon (IFN) trial, in which HRQoL for radically operated high-risk melanoma patients did not differ from that of the general population. The patients in the control arm not receiving IFN maintained their baseline levels of HRQoL [22]. Notably, patients in the IFN study were excluded if they had previous or ongoing depression, which was not the case in the TRIM trial. Nevertheless, these results give support to our findings. The same comparison was not possible for anxiety and depressive symptoms, since normative data are not available in this regard. Nevertheless, levels of anxiety and depression were generally low in the study group despite the participants’ surgery for invasive tumors with a significant risk of disease recurrence.

Our selection of oncology clinics means that differences in results between patients recruited at oncology as opposed to surgical centers cannot be ruled out. However, we assumed that the results would not differ whether patients underwent follow-up at surgical or oncological centers (which depended on geographical factors and local health care organizations), and patients selected for assessment of HRQoL and emotional problems were, therefore, enrolled at the major oncology centers that included the vast majority of all patients. Notably, 92% of the patients who were considered eligible for analysis answered the questionnaires at both baseline and 1 year, which indicates that our results are truly representative and reliable.

The TRIM study is still recruiting, and future analyses in more patients and with longer follow-up are planned. Whether the results presented in the current study will change when more data are added remains to be seen. However, since no clinically relevant differences could be detected at 1-year follow-up in this fairly large group of patients, there is presumably no major negative effect of imaging on patients´ HRQoL or anxiety and depressive symptoms just before the doctor´s appointment. It is possible that HRQoL, anxiety and depressive symptoms would have been rated differently if the assessments had taken place during the months between follow-up visits.

Another important issue that also is unclear, is whether our results might be generalized to follow-up programs in other cultural settings and for other types of cancer. A randomized study published in 1994, with similar aims as the TRIM study but regarding breast cancer, included over 1300 patients after surgery. The patients in the intensive surveillance group were followed up by imaging and blood tests in addition to clinical investigations and mammograms. Measurements of HRQoL were made at 6, 12, 24 and 60 months, and results were similar in both groups [23].

In conclusion, our results indicate that a follow-up schedule with whole-body imaging in high-risk melanoma patients does not cause clinically significant anxiety/depressive symptoms or have an impact on patients´ HRQoL. This is, to our knowledge, the first randomized study on the subject reported in melanoma patients. The results are important to consider in future decisions regarding follow-up guidelines for high-risk melanoma patients.

## Figures and Tables

**Table 1:** Patient characteristics.

Variable	Standard Arm (*n* = 105)	Experimental Arm (*n* = 99)
Sex		
Female	43 (41%)	36 (37%)
Male	62 (59%)	62 (63%)
Age median (IQR *)	67 (56, 73)	61 (50, 71)
Stage		
IIB	33 (31%)	24 (24%)
IIC	12 (11%)	13 (13%)
IIIA	16 (15%)	15 (15%)
IIIB-C	9 (9%)	11 (11%)
IIIB-D (incl T0)	31 (30%)	32 (32%)
IINX or III (tx)	4 (4%)	4 (4%)
Lymph node dissection performed	17 (16%)	14 (14%)
Post-operative treatment		
Radiotherapy	0 (0%)	3 (3%)
Systemic treatment	23 (22%)	19 (19%)

* Interquartile range.

**Table 2:** Mean scale scores including standard deviation (SD) for each subscale in QLQ-C30, HAD-A and HAD-D at 1 year.

Subscale	Standard Arm Mean Value and (SD)	Experimental Arm Mean Value and (SD)
Global health (QL)	79 (20)	77 (19)
Physical function (PF)	89 (17)	91 (13)
Role function (RF)	88 (22)	86 (25)
Emotional function (EF)	87 (15)	85 (19)
Cognitive function (CF)	89 (15)	88 (16)
Social function (SF)	91 (18)	89 (18)
Fatigue (FA)	18 (20)	20 (21)
Nausea and vomiting (NV)	1.4 (5.2)	3.7 (11)
Pain (PA)	15 (24)	13 (19)
Dyspnea (DY)	15 (23)	18 (24)
Insomnia (SL)	20 (27)	20 (29)
Loss of appetite (AP)	2.5 (11)	6.1 (19)
Constipation (CO)	6.7 (16)	8.4 (17)
Diarrhea (DI)	4.8 (12)	8.1 (18)
Financial difficulties related to disease (FI)	2.5 (11)	6.7 (19)
Anxiety (HAD-A)	3.1 (2.9)	3.9 (3.6)
Depression (HAD-D)	2.5 (2.7)	3.0 (2.9)

Anxiety and depression: Summarized HAD subscales (mean value). Missing data: 1 patient in experimental arm for dyspnea.

## Data Availability

The data presented in this study are available upon request to the corresponding author. The data are not publicly available due to patient safety and confidentiality requirements.
